# Analysis of Cation Composition in Dolomites on the Intact Particles Sampled from Asteroid Ryugu

**DOI:** 10.1021/acs.analchem.3c03463

**Published:** 2023-12-29

**Authors:** Mayu Morita, Hiroharu Yui, Shu-hei Urashima, Morihiko Onose, Shintaro Komatani, Izumi Nakai, Yoshinari Abe, Yasuko Terada, Hisashi Homma, Kazuko Motomura, Kiyohiro Ichida, Tetsuya Yokoyama, Kazuhide Nagashima, Jérôme Aléon, Conel M. O’D. Alexander, Sachiko Amari, Yuri Amelin, Ken-ichi Bajo, Martin Bizzarro, Audrey Bouvier, Richard W. Carlson, Marc Chaussidon, Byeon-Gak Choi, Nicolas Dauphas, Andrew M. Davis, Wataru Fujiya, Ryota Fukai, Ikshu Gautam, Makiko K. Haba, Yuki Hibiya, Hiroshi Hidaka, Peter Hoppe, Gary R. Huss, Tsuyoshi Iizuka, Trevor R. Ireland, Akira Ishikawa, Shoichi Itoh, Noriyuki Kawasaki, Noriko T. Kita, Kouki Kitajima, Thorsten Kleine, Sasha Krot, Ming-Chang Liu, Yuki Masuda, Frédéric Moynier, Ann Nguyen, Larry Nittler, Andreas Pack, Changkun Park, Laurette Piani, Liping Qin, Tommaso Di Rocco, Sara S. Russell, Naoya Sakamoto, Maria Schönbächler, Lauren Tafla, Haolan Tang, Kentaro Terada, Tomohiro Usui, Sohei Wada, Meenakshi Wadhwa, Richard J. Walker, Katsuyuki Yamashita, Qing-Zhu Yin, Shigekazu Yoneda, Edward D. Young, Ai-Cheng Zhang, Tomoki Nakamura, Hiroshi Naraoka, Takaaki Noguchi, Ryuji Okazaki, Kanako Sakamoto, Hikaru Yabuta, Masanao Abe, Akiko Miyazaki, Aiko Nakato, Masahiro Nishimura, Tatsuaki Okada, Toru Yada, Kasumi Yogata, Satoru Nakazawa, Takanao Saiki, Satoshi Tanaka, Fuyuto Terui, Yuichi Tsuda, Sei-ichiro Watanabe, Makoto Yoshikawa, Shogo Tachibana, Hisayoshi Yurimoto

**Affiliations:** † Analytical Technology Division, Horiba Techno Service Co., Ltd., Kyoto 601-8125, Japan; ‡ Department of Chemistry, Tokyo University of Science, Tokyo 162-8601, Japan; § Department of Applied Chemistry, Tokyo University of Science, Tokyo 162-8601, Japan; ∥ Graduate School of Engineering Materials Science and Engineering, Tokyo Denki University, Tokyo 120-8551, Japan; ⊥ Spectroscopy and Imaging Division, Japan Synchrotron Radiation Research Institute, Hyogo 679-5198, Japan; # Osaka Application Laboratory, Rigaku Corporation, Osaka 569-1146, Japan; ¶ Thermal Analysis Division, Rigaku Corporation, Tokyo 196-8666, Japan; ∇ Department of Earth and Planetary Sciences, Tokyo Institute of Technology, Tokyo 152-8551, Japan; ○ Hawai‘i Institute of Geophysics and Planetology, University of Hawai‘i at Ma̅noa, Honolulu, Hawaii 96822, United States; ⧫ Institut de Minéralogie, de Physique des Matériaux et de Cosmochimie, Sorbonne Université, Museum National d’Histoire Naturelle, Centre National de la Recherche Scientifique Unité Mixte de Recherche 7590, Institut de recherche pour le développement, Paris 75005, France; †† Earth and Planets Laboratory, Carnegie Institution for Science, Washington, District of Columbia 20015, United States; ‡‡ McDonnell Center for the Space Sciences and Physics Department, Washington University, St. Louis, Missouri 63130, United States; §§ Geochemical Research Center, The University of Tokyo, Tokyo 113-0033, Japan; ∥∥ Guangzhou Institute of Geochemistry, Chinese Academy of Sciences, Guangzhou, GD 510640, China; ⊥⊥ Department of Natural History Sciences, Hokkaido University, Sapporo 001-0021, Japan; ## Centre for Star and Planet Formation, Globe Institute, University of Copenhagen, Copenhagen K 1350, Denmark; ¶¶ Bayerisches Geoinstitut, Universität Bayreuth, Bayreuth 95447, Germany; ∇∇ Université Paris Cité, Institut de physique du globe de Paris, Centre National de la Recherche Scientifique, Paris 75005, France; ○○ Department of Earth Science Education, Seoul National University, Seoul 08826, Republic of Korea; ⧫⧫ Department of the Geophysical Sciences and Enrico Fermi Institute, University of Chicago, Chicago, Illinois 60637, United States; ††† Faculty of Science, Ibaraki University, Mito 310-8512, Japan; ‡‡‡ Institute of Space and Astronautical Science (ISAS), Japan Aerospace Exploration Agency (JAXA), Sagamihara 252-5210, Japan; §§§ Department of General Systems Studies, University of Tokyo, Tokyo 153-0041, Japan; ∥∥∥ Department of Earth and Planetary Sciences, Nagoya University, Nagoya 464-8601, Japan; ⊥⊥⊥ Max Planck Institute for Chemistry, Mainz 55128, Germany; ### Department of Earth and Planetary Science, University of Tokyo, Tokyo 113-0033, Japan; ¶¶¶ School of Earth and Environmental Sciences, University of Queensland, St Lucia, QLD 4072, Australia; ∇∇∇ Division of Earth and Planetary Sciences, Kyoto University, Kyoto 606-8502, Japan; ○○○ Department of Geoscience, University of Wisconsin—Madison, Madison, Wisconsin 53706, United States; ⧫⧫⧫ Max Planck Institute for Solar System Research, Göttingen 37077, Germany; †††† Department of Earth, Planetary, and Space Sciences, University of California, Los Angeles, California 90095, United States; ‡‡‡‡ Astromaterials Research and Exploration Science Division, National Aeronautics and Space Administration Johnson Space Center, Johnson Space Center, Houston, Texas 77058, United States; §§§§ Faculty of Geosciences and Geography, University of Göttingen, Göttingen D-37077, Germany; ∥∥∥∥ Division of Earth-System Sciences, Korea Polar Research Institute, Incheon 21990, Korea; ⊥⊥⊥⊥ Centre de Recherches Pétrographiques et Géochimiques, Centre National de la Recherche Scientifique-Université de Lorraine, Nancy 54500, France; #### School of Earth and Space Sciences, University of Science and Technology of China, Anhui 230026, China; ¶¶¶¶ Department of Earth Sciences, Natural History Museum, London SW7 5BD, U.K.; ∇∇∇∇ Isotope Imaging Laboratory, Hokkaido University, Sapporo 001-0021, Japan; ○○○○ Institute for Geochemistry and Petrology, Department of Earth Sciences, ETH Zurich, Zurich 8092, Switzerland; ⧫⧫⧫⧫ Department of Earth and Space Science, Osaka University, Osaka 560-0043, Japan; ††††† School of Earth and Space Exploration, Arizona State University, Tempe, Arizona 85281, United States; ‡‡‡‡‡ Department of Geology, University of Maryland, College Park, Maryland 20742, United States; §§§§§ Graduate School of Natural Science and Technology, Okayama University, Okayama 700-8530, Japan; ∥∥∥∥∥ Department of Earth and Planetary Sciences, University of California, Davis, California 95616, United States; ⊥⊥⊥⊥⊥ Department of Science and Engineering, National Museum of Nature and Science, Tsukuba 305-0005, Japan; ##### School of Earth Sciences and Engineering, Nanjing University, Nanjing 210023, China; ¶¶¶¶¶ Department of Earth Science, Tohoku University, Sendai 980-8578, Japan; ∇∇∇∇∇ Department of Earth and Planetary Sciences, Kyushu University, Fukuoka 819-0395, Japan; ⧫⧫⧫⧫⧫ Earth and Planetary Systems Science Program, Hiroshima University, Higashi-Hiroshima 739-8526, Japan; †††††† Graduate School of Engineering, Kanagawa Institute of Technology, Atsugi 243-0292, Japan; ‡‡‡‡‡‡ UTokyo Organization for Planetary and Space Science (UTOPS), University of Tokyo, Tokyo 113-0033, Japan

## Abstract

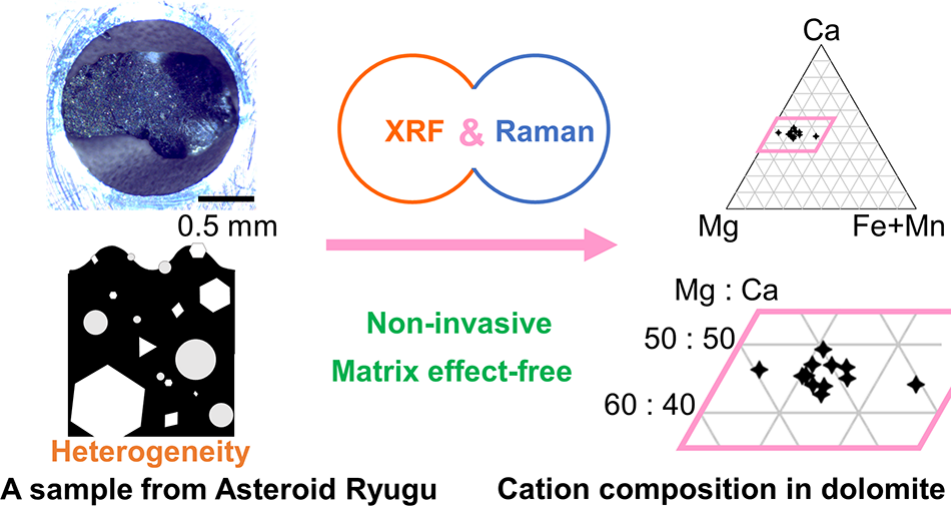

Characterization of the elemental distribution of samples with rough surfaces has been strongly desired for the analysis of various natural and artificial materials. Particularly for pristine and rare analytes with micrometer sizes embedded on specimen surfaces, non-invasive and matrix effect-free analysis is required without surface polishing treatment. To satisfy these requirements, we proposed a new method employing the sequential combination of two imaging modalities, i.e., microenergy-dispersive X-ray fluorescence (micro-XRF) and Raman micro-spectroscopy. The applicability of the developed method is tested by the quantitative analysis of cation composition in micrometer-sized carbonate grains on the surfaces of intact particles sampled directly from the asteroid Ryugu. The first step of micro-XRF imaging enabled a quick search for the sparsely scattered and micrometer-sized carbonates by the codistributions of Ca^2+^ and Mn^2+^ on the Mg^2+^- and Fe^2+^-rich phyllosilicate matrix. The following step of Raman micro-spectroscopy probed the carbonate grains and analyzed their cation composition (Ca^2+^, Mg^2+^, and Fe^2+^ + Mn^2+^) in a matrix effect-free manner via the systematic Raman shifts of the lattice modes. The carbonates were basically assigned to ferroan dolomite bearing a considerable amount of Fe^2+^ + Mn^2+^ at around 10 atom %. These results are in good accordance with the assignments reported by scanning electron microscopy–energy-dispersive X-ray spectroscopy, where the thin-sectioned and surface-polished Ryugu particles were applicable. The proposed method requires neither sectioning nor surface polishing; hence, it can be applied to the remote sensing apparatus on spacecrafts and planetary rovers. Furthermore, the non-invasive and matrix effect-free characterization will provide a reliable analytical tool for quantitative analysis of the elemental distribution on the samples with surface roughness and chemical heterogeneity at a micrometer scale, such as art paintings, traditional crafts with decorated shapes, as well as sands and rocks with complex morphologies in nature.

## Introduction

The analysis of the elemental distribution is fundamental for characterizing natural and artificial materials. However, these materials often have surface roughness and a complicated structure. Examples are environmentally polluting substances with heavy metal elements, geo- and cosmo-chemical samples with microcrystals of minerals, artworks and sculptures with surface paintings, and fragments left on crime scenes.

For the quantitative analysis of the elemental composition of materials, there are several bulk analytical techniques that give precise values, such as inductively coupled plasma mass spectroscopy (ICP–MS).^1^ However, heterogeneous characteristics such as surface roughness and chemical heterogeneity of the sample will be deprived by its dissolution procedures. The obtained elemental composition averages the entire volume sampled in the case of destructive analyses.

For the analysis of elemental distributions of the sample surfaces, scanning electron microscopy with energy dispersive X-ray spectroscopy (SEM–EDS), electron probe microanalyzer (EPMA), and secondary ion mass spectrometry (SIMS)^1^ are powerful analytical tools. Their imaging modalities enable us to measure such physically and chemically heterogeneous samples with excellent spatial resolution at a micrometer-to-nanometer scale. However, they generally require pretreatments such as sample sectioning, surface polishing, or metal/carbon coating to avoid charging due to the irradiation of electron or ion beams onto the sample surfaces. These pretreatments sometimes lose information about surface roughness or porosity and affect the accuracy of quantitative estimations of the spatially distributed elements. Accordingly, a non-invasive analytical method is strongly desired to evaluate the spatial distributions of elements with their surface morphology intact. Here, “non-invasive” is defined as a procedure not involving irreversible alternations by physical or chemical processes in the pretreatments, such as surface polishing or metal/carbon coating.

Microenergy-dispersive X-ray fluorescence (micro-XRF) allows us to visualize the heterogeneous spatial distribution of elements at the micrometer scale without any pretreatments.^2^ Synchrotron radiation micro-XRF (SR-XRF)^3^ is of particular advantage as a high-energy source with strong brightness and excellent spatial resolution at a submicron scale. Furthermore, X-ray absorption fine structure (XAFS) can be simultaneously obtained with SR-XRF, which enables us to reveal the chemical state of that sample. One notable disadvantage in SR-XRF is its quite limited access to synchrotron light sources. Alternatively, laboratory micro-XRF offers much easier access. However, the primary X-rays focused by a glass capillary result in a spatial resolution typically of 100 μm or, at best, 10 μm. When the size of the analytes of interest is on the micrometer scale, these spatial resolutions induce another drawback: the unwanted overlap of signals from the matrix surrounding the micrometer-sized analytes. Furthermore, compared to an electron or ion beam, an X-ray penetrates further inside the analyte. Therefore, when the analyte is thin, the detection area also expands into the depth direction of the matrix. Consequently, three-dimensional signal overlap reduces the accuracy of the quantitative estimation of elements in the aimed analyte. In the present paper, these problems, originating from both the instrumental limitations of micro-XRF and sample size and morphology, are all referred to as “matrix effects”.

Another candidate is Raman micro-spectroscopy. It is also a non-invasive analytical technique that provides information on slight changes in molecular or crystal structures with superior lateral spatial resolution (normally 1 μm) to that of micro-XRF. Furthermore, since the spectral patterns of Raman scattering originate from molecular or crystal structures, we can discriminatively measure these analytes in the focused area. In other words, we can distinguish Raman signals of the targeted molecules and crystals selectively from those of the matrix. However, Raman micro-spectroscopy also has disadvantages in measurement time due to its inherently weak signal. Thus, the imaging area is quite limited by trading off its spatial resolution. This disadvantage could be serious when the analyte is rare and is heterogeneously and sparsely scattered on the surface of the specimen on a millimeter or even larger scale.

To overcome these difficulties, we propose a new analytical method of micro-XRF and Raman micro-spectroscopy. To examine the capability of the proposed method, we studied the samples directly collected from the asteroid Ryugu by the Hayabusa2 spacecraft^4,5^ as a practical example of an extremely rare, heterogeneous, and complex sample with rugged surfaces. They have various micrometer-scaled grains of minerals sparsely existing on the surface of the phyllosilicate matrix.^6^

Among the various minerals found on the Ryugu samples, we focused on carbonates. This is because they are important secondary minerals formed by aqueous alteration and are expected to provide quite fruitful information on the aqueous environments where they were formed in the early solar system.^7,8^ In terrestrial environments, they are commonly found in sedimentary rocks. Their chemical characteristics and cation compositions help us understand the chemical histories of the atmosphere, oceans, and climate change that they experienced.^9^ Furthermore, interestingly, the kinds of carbonate have many variations depending on cation compositions, e.g., calcite, magnesite, dolomite, and siderite.^9^ Some trace or minor elements that preferentially occur in carbonate grains are manganese, bromine, strontium, and yttrium.^9^ Among these elements, the magnesium ion in dolomite can be easily substituted by manganese or iron. In particular, previous studies reported that this substitution frequently occurred on the dolomites of the most primitive meteorites that were formed during the early stages of the solar system.^10−12^

In recent studies, we have developed scales to quantitatively evaluate the degree of cation substitution of carbonates that occurred on natural dolomites using peak shifts of Raman frequencies.^13−15^ Since natural dolomite forms dolomite [CaMg(CO_3_)_2_]–ankerite [CaFe(CO_3_)_2_]_3_ solid solution systems,^16,17^ the quantitative estimation of Fe^2+^(+Mn^2+^) is important to investigate the chemical environment where they occurred. The present study demonstrates that micro-XRF imaging realizes the efficient search of dolomite crystals on the Ryugu particles, and that the following Raman imaging at the region of interest (ROI) area can estimate the cation composition quantitatively without matrix effects. The proposed method is sufficiently pragmatic to quantitatively estimate the cation compositions of Ryugu dolomites in a matrix effect-free and non-invasive manner.

## Experimental Section

### Elemental Imaging by Micro-XRF

Micro-XRF was performed at HORIBA X-ray LAB with version 1.3.2.19 software (XGT-9000, HORIBA Co., Ltd.). An arrayed multichamber cell (AMCC; see the following section for the details) was loaded into the measurement position under vacuum conditions. The primary X-rays were focused to 100 μm in diameter with a glass polycapillary optic and vertically irradiated the sample. The fluorescence X-rays from the samples were measured by a silicon drift EDS detector at an angle of 45°. An Rh target X-ray tube was used to generate the primary X-rays. A specimen stage in the XGT-9000 scanned the sample position, while obtaining a spectrum step by step. While the scanning step was slightly varied depending on the desired imaging area, it was typically 14 μm and, at its largest, 20 μm. A step size smaller than the beam size (i.e., spatial resolution) was chosen for definitely finding the Ca-rich spots. Namely, the XRF beam was not spatially homogeneous, and hence, the center of the spot tends to be preferentially probed. Conversely, if the step size was comparable to the beam size, small Ca-rich spots located just between the focused spots might accidentally fail to be found because of the low X-ray photon density at the edge of the beam. The step size being sufficiently smaller than the beam size helps dispel such concerns. By processing the spectrum and calculating the X-ray intensity of an element, the distribution of the element was visualized; this process is called elemental imaging or micro-XRF imaging in the present paper. The measurement conditions of the X-ray tube voltage, the measurement time, and X-ray working distance were 30 kV, 200 ms per pixel, and 1.0 mm, respectively. The tube current was set to 300 μA.

### Raman Micro-spectroscopy

Micro-Raman measurements were performed with LabRAM HR Evolution and LabSpec 6 software (HORIBA Co., Ltd.). For the detector, a Synapse EMCCD camera (HORIBA Co., Ltd.) was equipped. The confocal optical arrangement of the microscope and autofocusing stage-driving with the software enabled us to obtain Raman mapping data with high spatial resolution even from the samples with surface roughness and without any pretreatment, such as surface polishing. Olympus LMPlanFN (100x, NA 0.80, WD 3.4 mm) was used as an objective lens for high-resolution Raman mapping with (sub)micrometer resolution. The spatial resolution (focus size of the laser) would be 0.4 μm at best under an assumption that the beam size of the incident light perfectly matched the pupil diameter of the objective lens. While it is difficult to prove that the size matching is perfect, the spot size (i.e., the spatial resolution) should not far exceed 1 μm. The excitation wavelength and power were 532 nm and 0.6–1.2 mW, respectively. The spectra were measured with 120 s exposure and averaged 2 times. The Raman signal from a Si wafer (520.6 cm^–1^) and a sulfur flake (153.8, 219.1, and 473.2 cm^–1^) was used for calibrating the Raman wavenumber.

### Estimating Cation Composition and Construction of the Ternary Diagram

To determine the cation compositions for terrestrial carbonates, the standard fundamental parameter (FP) method^18,19^ was applied to the XRF spectra. The cation compositions were calibrated by standard samples named JLk-1 and Dolomite (MV) for kutnohorite^14^ and dolomite, respectively. The standard FP method can calibrate fundamental parameters, including coefficients for elemental intensity, stored in the instrumentation, which are subject to matrix effects. JLk-1 is lake sediment from Lake Biwa, Shiga, Japan, which is a geostandard sample provided by the National Institute of Advanced Industrial Science and Technology (AIST), Japan. Dolomite (MV) is a dolomite stone mined in Morro Velho, Brazil. A single crystal (0.1697 g) of Dolomite (MV) was used as the standard, and its cation composition was determined by inductively coupled plasma-atomic emission spectroscopy (ICP-AES). The details of the ICP-AES experiment can be found in the Supporting Information (SI). Each cation content was finally given by the simple eq 1 below.
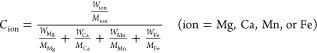
1*C*_ion_: cation content [atom %], *W*_ion_: elemental concentration [wt %], and *M*_ion_: atomic weight.

### Arrayed Multi-chamber Cell

To implement two measurements of micro-XRF and Raman micro-spectroscopy sequentially, we have developed a sample holder called AMCC (Figure S1 in the Supporting Information). Specifically, we drilled holes 0.5 mm deep with diameters ranging from 0.5 to 1.2 mm at 0.1 mm pitch (Figure S1a–c). When loading the sample(s), we carefully chose a hole that would fit the size of the Ryugu particles. This is because the Ryugu particles might be lost in the instrument chambers due to air flowing upon vacuuming or due to static electricity. When the AMCC was placed on the specimen stage of the respective microscope, AMCC jigs were used (Figure S1d,f). During the micro-XRF measurement, the pressure in the sample chamber was simultaneously monitored to keep it below tens of Pa. When transferring the AMCC from micro-XRF to a Raman microscope, the pressure in the sample chamber was gradually released to atmospheric pressure, and the AMCC was carefully detached from the stage to reduce the risk of sample loss. When the sample amount is as small as or less than 1 mg, the holes may be unfit due to static electricity. Therefore, a 0.5 mm thick aluminum cell frame was set to load a single Ryugu particle into a hole with an appropriate diameter. As for measuring the reference minerals, they were fixed by acrylic cell frames. Note that the reference minerals were large enough not to worry about the risk of sample loss.

### Samples

A Ryugu particle picked up from the A0107 aggregate sample collected at the first touchdown site (TD1) was measured in this study.^5^

The mined locations of the geostandard and terrestrial carbonates are summarized as follows. JLk-1 is from Lake Biwa, Shiga, Japan. Calcite is from Garo, Hokkaido, Japan. Magnesite is from Goat Hill Magnesia Quarries, Pennsylvenia, USA. Kutnohorite (SH) is from Sterling Mine, New Jersey, USA. Kutnohorite (W) is from Wissels Mine, Northern Cape, South Africa. Dolomite (MV) is from Morro Velho Mine, Minas Gerais, Brazil. Ferroan dolomite (EM) is from Eagle Mine, Colorado, USA. Their optical images are shown in Figure S2 in the Supporting Information.

## Results and Discussion

### Mn–Ca Colocalization on the Ryugu Particle

**Figure 1:**
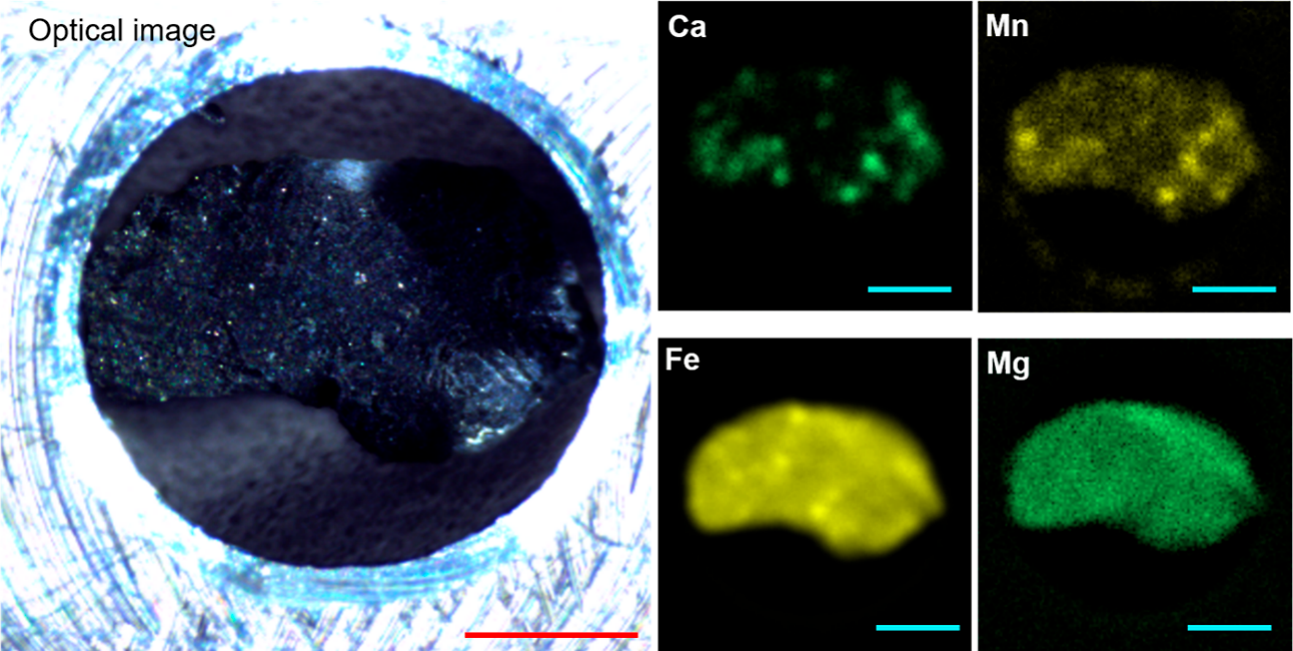
Optical image of the Ryugu particle in AMCC (left, scale bars: 0.5 mm) and the corresponding elemental images for Ca, Mn, Fe, and Mg (four panels in right, scale bars: 0.5 mm).

Figure 1 shows the elemental imaging of the Ryugu particle (from A0107) with micro-XRF. As shown in Figure 1, Mg and Fe were predominantly present. In contrast, the distribution of Ca was sparse and heterogeneous. Mn also showed a spatial distribution similar to that of Ca; the bright spots of Mn overlapped well with those of Ca. Based on previous studies,^10−12^ this spatial matching of Ca and Mn strongly suggests that these spots correspond to the positions where carbonate grains exist. The other two major cations in carbonates, i.e., Mg and Fe, are also abundantly included in the matrix of phyllosilicate minerals. Therefore, elemental images of Ca and Mn help us find the locations of carbonate grains on the matrix. However, since the carbonates are identified with Ca, Mg, and Fe + Mn, it is hard to identify carbonate species based only on Ca and Mn distribution.

In Figure 1, it should be noted that not only the bright spots of Ca and Mn match spatially well but also the brightness of each element varies depending on the locations. This result implies that there are a variety of cation compositions in each carbonate grain at the Ca–Mn colocalization sites. However, we must remark that the brightness variation might simply derive from the matrix effects, which indicates the difficulty in estimating cation compositions merely through micro-XRF.

### Raman Spectra Measured at Ca–Mn-Rich Spots

**Figure 2:**
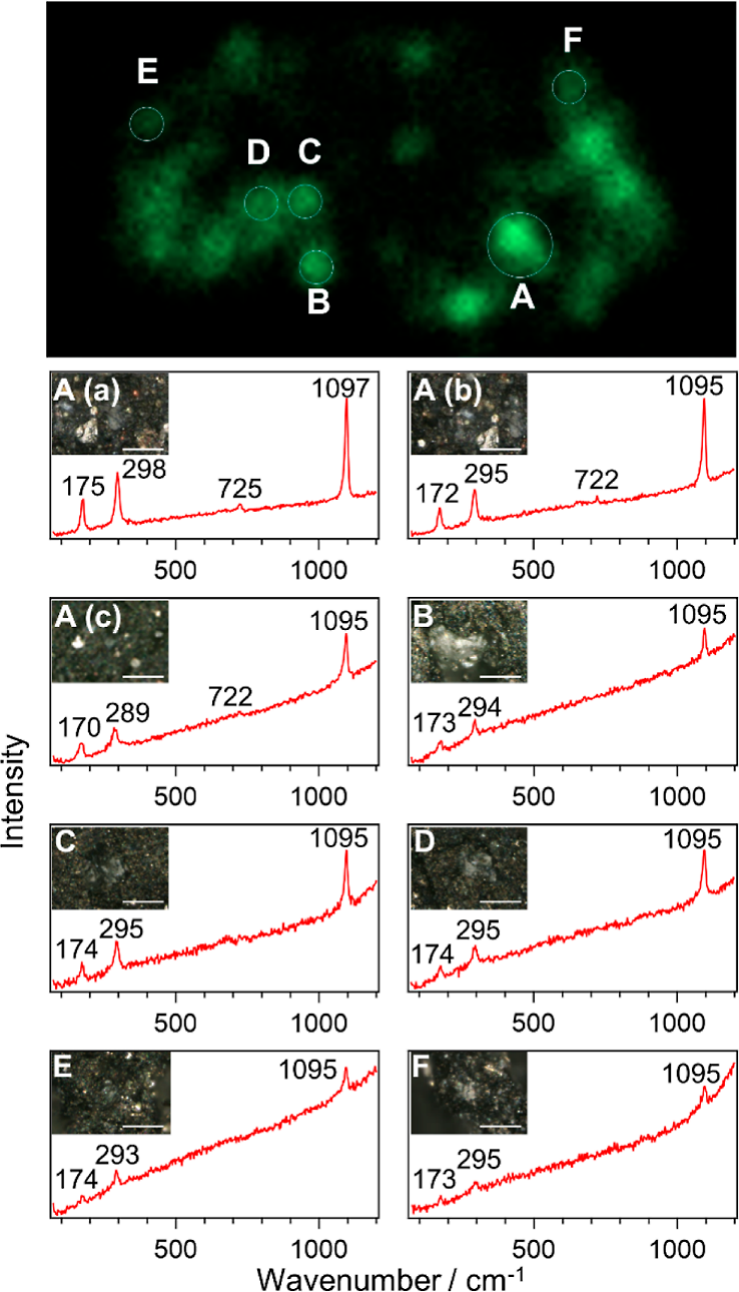
Ca bright spots on the surface of the Ryugu particle by micro-XRF imaging and their corresponding Raman spectra by a Raman microscope. The inset in each spectrum is the corresponding optical image (scale bars: 20 μm). Grayish grains on black matrices are carbonates.

To confirm whether carbonate grains actually exist at the Ca–Mn colocalization spots, we then measured Raman spectra at the Ca–Mn rich spots appearing in the top panel of Figure 2. Note that probing the identical spots for micro-XRF and Raman microscopes was achieved through the use of the identical AMCC mentioned above. It is also noteworthy that from optical microscopic images, i.e., without the help of the elemental map observed by XRF, carbonate grains on Ryugu as well as on other meteorites are generally indistinguishable from those of other minerals such as silicates and iron oxides. As shown in Figure 2, three or four sharp peaks with a broad fluorescence background appeared in the Raman spectra at all measured colocalization spots. The peak wavenumbers for these bands were around 174, 295, 724, and 1094 cm^–1^, as summarized in Table 1. (Note that, strictly speaking, the “wavenumber” should be referred to as the relative wavenumber to that of incident light or conventionally “Raman shift”. In the present paper, however, we use “wavenumber” because the words “relative” and “shift” have started to be avoided in recent years, presumably as they make an impression that the wavenumber can change due to some perturbations such as temperature and pressure changes.) These bands are characteristic to carbonates, and each peak is assignable to translational lattice mode (T), librational lattice mode (L), CO_3_^2–^ bending mode (ν_4_), and CO_3_^2–^ stretching mode (ν_1_), respectively.^20,21^ The appearance of the set of these peaks ensures that there are indeed carbonate grains at all of the Ca–Mn colocalization spots. Furthermore, the peak wavenumbers are known to shift, depending on the cation compositions in the carbonates. By comparing the peak wavenumbers with those observed in previous studies,^13−15^ we assigned these carbonates essentially to dolomite CaMg(CO_3_)_2_. Note that Mg in dolomite is often partially substituted by Fe or Mn, forming dolomite [CaMg(CO_3_)_2_]–ankerite [CaFe(CO_3_)_2_]–kutnohorite [CaMn(CO_3_)_2_] solid solution series.^16,17^ In fact, the peak wavenumbers summarized in Table 1 slightly deviated from each other, implying different degrees of cation substitution occurring at each carbonate grain. The slightly lower wavenumbers of the ν_1_ mode compared to that of terrestrial dolomite would be also due to the substitution. The cation substitution will be quantitatively discussed in detail in the following section.

**Table 1:** Raman Frequencies for Ryugu Carbonate Grains and Terrestrial References (unit: cm^–1^)

	T	L	ν_4_	ν_1_
A(a)	176.4	298.9	724.9	1096.8
A(b)	173.7	296.0	721.7	1094.8
A(c)	171.6	289.7	722.3	1095.0
B	173.8	295.0		1095.3
C	175.0	296.1		1095.6
D	175.1	296.3		1095.0
E	175.0	294.6		1094.6
F	174.5	296.1		1094.7
calcite	154.6 ± 0.9	280.9 ± 0.9	711.6 ± 0.8	1086.0 ± 0.8
magnesite	212.5 ± 1.0	329.3 ± 1.0	738.2 ± 1.1	1094.4 ± 1.0
Dolomite (MV)	174.1 ± 0.2	296.7 ± 0.2	724.3 ± 0.1	1096.8 ± 0.2

### Estimation of Fe + Mn Content in Ryugu Dolomites

The partial substitution of Mg for Fe or Mn was suggested by both micro-XRF and Raman microspectroscopy. However, since Mg and Fe exist everywhere in the matrix, it is difficult to selectively quantify the amount of these cations in carbonates solely with micro-XRF. On the other hand, in our previous studies, it was found that the cation composition of carbonates can be uniquely determined from their Raman wavenumbers of T- and L-modes within an error of 1.7%.^14,15^ While a couple of conversion equations were proposed in the previous studies,^13−15,22−27^ it is essential to first qualitatively identify the type of carbonates (i.e., major cations contained) in order to choose the most suitable equation.

**Figure 3:**
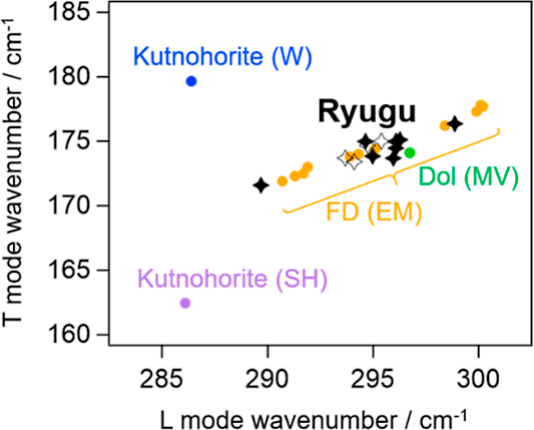
Biaxial plotting and comparison for Ryugu dolomites and terrestrial references with T- and L-mode wavenumbers. For Ryugu dolomites, the black filled markers represent those obtained at grains A(a)-F, while the open markers represent those obtained at several different spots within grain B. Dol stands for dolomite, and FD stands for ferroan dolomite.

For this purpose, the T- and L-mode frequencies of Ryugu carbonates are biaxially plotted together with those of terrestrial carbonates belonging to the dolomite-ankerite-kutnohorite solid solution series (Figure 3). In Figure 3, two kurnohorites labeled (SH) and (W), which were collected at Sterling Hills (USA) and Wissels Mine (South Africa), respectively, are shown because their cation compositions were noticeably different (XRF spectra can be found in Figure S3 in the Supporting Information). A wide distribution of the T- and L-mode wavenumbers found for terrestrial ferroan dolomite [Eagle Mine (EM), USA; data taken from ref (13)] is due to heterogeneity of cation composition in a sample. The cation compositions of the terrestrial carbonates are summarized in Table S1 in the Supporting Information. By comparison of the results for terrestrial and Ryugu carbonates, Ryugu carbonates are assignable to ferroan dolomite, while their wavenumbers also slightly depend on the measurement position, reflecting their heterogeneity.

As already reported in our previous studies,^13,14^ the cation compositions of ferroan dolomites can be obtained by the T- and L-mode wavenumbers. For *C*_Fe+Mn_, it is
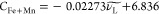
2where  is the L-mode wavenumber in inverse centimeters.^13^ For *C*_Ca_, although we had reported a similar equation (),^14^ here it is revised as
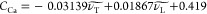
3where  is the T-mode wavenumber in inverse centimeters. The reason for this revision is that the Ca content in a standard sample was overestimated in the previous study.^14^ The coefficients of eq 3 were derived in the same way as those in the previous study, but a more reliable standard was used here by changing the reference dolomite from JDo-1 (dolostone) to a dolomite crystal (Morro Velho Mine, Brazil). The details of the derivation of eq 3 and the reason for the modification can be found in SI with a Raman spectrum of JDo-1 (Figure S4). We should remark that this revision decreases the calculated Ca content by about 8 ± 2 atom % from those calculated by the original one. Note that the reason for the overestimation of Ca content in the previous equation is that JDo-1 contains a noticeable amount of calcite, even though it is provided as a “dolomite rock”. Namely, while *C*_Ca_ was provided as 0.57 by the public organization AIST, some of the Ca originates from calcite, not dolomite. Although it is quite difficult to obtain *C*_Ca_ of the dolomite portion in JDo-1 because it is a powder mixture of dolomite and calcite, by assuming it is 0.50 such as in the ideal case of dolomite CaMg(CO_3_)_2_, the previous equation should overestimate *C*_Ca_ about 0.07 (7 atom %). The coincidence between this rough estimation (7 atom %) and the experimental results (8 ± 2 atom %) supports that the revised equation is more reliable. By determining *C*_Ca_ and *C*_Fe+Mn_, *C*_Mg_ can be derived from eq 1, namely, *C*_Ca_ + *C*_Mg_ + *C*_Fe+Mn_ = 1. One may concern oneself with the reliability and the significant digits in eqs 2 and 3. Our previous study demonstrated that the error of the equations is 0.04 (4 atom %) at worst. Therefore, the last digits of the composition obtained by the equations should be on the order of 0.01. Because the rounding should be taken after the summation, the significant digits of the coefficients were determined for the last digits of each term of the right hand in eqs to be 0.001. Note that the accuracy of the peak wavenumber in the present study was typically around 0.2 cm^–1^, and it gives rise to a composition error of less than 1 atom % according to the error propagation theory. Therefore, the composition error due to the Raman measurement would be negligible as long as the Raman spectra are properly measured.

**Figure 4:**
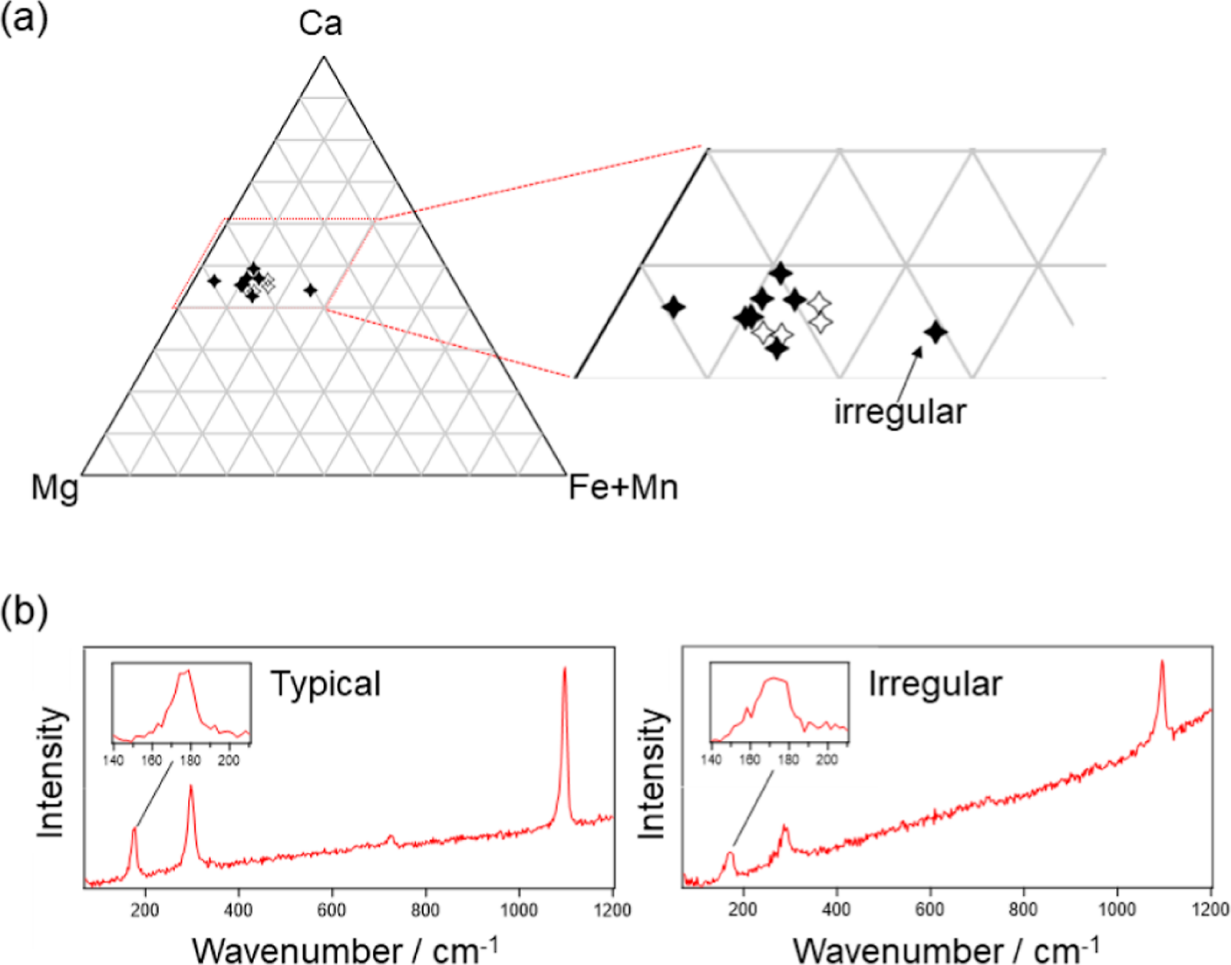
Cation composition for Ryugu dolomites. (a) Cation compositions of Ryugu dolomites plotted on a ternary diagram. The filled and open markers represent those obtained at the spot A(a) to F and those within the spot B, respectively. (b) Raman spectra measured at the spots showing typical and irregular compositions. “Typical” spectrum is of the spot A(a) while “Irregular” is of the spot A(c).

The cation compositions of Ryugu dolomites derived by eqs 1−3 are plotted in the ternary diagram (Figure 4a). The results indicate that the majority of Ryugu dolomites contain about 10–15% Fe or Mn. In a recent study, a ternary diagram (Ca, Mg, and Fe + Mn) of Ryugu dolomites reported with SEM–EDX measurements showed on average 10–20% for *C*_Fe+Mn_.^28,29^

The distribution is in good accordance with that in the present study. This consistency ensures the reliability of the proposed analytical method. Furthermore, the present method does not require pretreatments of samples, such as surface polishing. It is worth highlighting that the quantitative estimation was achieved in the totally non-invasive manner without matrix effects.

For the grain whose Fe + Mn composition is about 25% (the one labeled “Irregular” in Figure 4a), it should be noted that the Raman peaks obtained at the spot [A(c)] were significantly broader than those typically obtained. The reason for the band broadening is unclear, but a plausible explanation is the low crystallinity of the dolomite grain at this spot. Because it is difficult to determine the peak position for the broad bands, it cannot be concluded currently whether the irregularly high concentration (∼25%) of Fe and Mn was true or just an artifact due to the broadened Raman band. It is worth keeping in mind that an adequate quality of the spectra (i.e., enough peak intensity and the sharpness of the peak without irregular broadening) is necessary for the estimation of accurate composition. This point is essential, especially when this technique is automatically applied to Raman mapping data. While the automatic analysis may provide a peak wavenumber even if the spectral quality is not sufficient, its reliability should be carefully checked by the signal-to-noise ratio, signal-to-background ratio, and sharpness of the bands. It is also unsatisfactory that Fe and Mn are indistinguishable due to their similarity in ionic radii and masses.^13,14^ However, it is worth noting that such quantitative analysis is achieved not by signal intensity but by the Raman shifts of the signals. As Raman shifts are influenced by neither the matrix effect nor the total amount of the sample, the composition can be accurately estimated.

As mentioned above, carbonate grains are crucial as evidence of experienced aqueous alteration, and the heterogeneity of cation abundance should provide important information to infer the aqueous environment. In the present paper, the elemental imaging of micro-XRF enhances efficient searching of sparsely distributed carbonates without pretreatments such as surface polishing and metal/carbon coating. Thanks to the sample selectivity and characteristic shifts of Raman microspectroscopy, it is also advantageous to quantitatively estimate the heterogeneity of cation composition in the same carbonate grain simultaneously. Raman spectra at a few spots within spot B were measured (Figure S5 in the Supporting Information) and analyzed, and it turned out that the cation compositions were heterogeneous even inside a single dolomite grain, as shown in Figures 3 and 4a. The proposed method, based on the sequential combination of XRF and Raman microscopes, would provide an initial analysis tool for evaluating the microheterogeneity of rare and pristine samples with surface roughness without any pretreatments.

## Summary

The sequential analysis of micro-XRF and Raman spectroscopy on the surfaces of Ryugu intact particles at the same measurement positions revealed that the Ca–Mn colocalized spots were found to be carbonate grains, and most of them can be assigned to ferroan dolomites, where the Fe–Mn ratio was estimated to be around 10–15%. These results indicate that the Ryugu dolomites have features similar to those found on CI-class meteorites, where Fe and Mn are considerably included. From an analytical point of view, we demonstrated that the sequential analysis of micro-XRF and Raman spectroscopy greatly enhanced finding and quantitatively characterizing important minerals that sparsely existed at the micrometer scale on the sample surfaces. The non-invasive manner of these two spectroscopies does not require pretreatments such as surface polishing before measurements. These points are favorable for the initial analysis of such extremely rare samples before performing further precise but invasive or destructive analyses to grasp the general features of the analytes and enhance the reliability of quantitative data estimated by other conventional methods. Moreover, a noteworthy advantage of the proposed method is that it provides a versatile tool to demonstrate the microregion heterogeneity of composition, while avoiding any matrix effects due to sample characteristics which are sometimes problematic in analyses utilizing X-rays. The proposed analytical approach is also applicable to a variety of samples of artwork and geo- or cosmo-chemical specimens.

## Supporting Information Available

Experimental details of ICP-AES and micro XRF, derivation of eq 3, cation compositions for terrestrial carbonates, detailed pictures of AMCC itself and samples loaded on AMCC, XRF spectra of the terrestrial samples, and Raman spectrum of JDo-1 (PDF)
