# Supplementary material for: Analysis of Cation Composition in Dolomites on the Intact Particles Sampled from Asteroid Ryugu
Source: Anal Chem. 2023 Dec 29;96(1):170–8. doi: 10.1021/acs.analchem.3c03463 (PMC10783172; doi:10.1021/acs.analchem.3c03463)
Supplement: Supplementary file 1 — ac3c03463_si_001.pdf [file ac3c03463_si_001.pdf]

Supporting Information for:

Analysis of Cation Composition in Dolomites on the Intact Particles Sampled from Asteroid Ryugu

Mayu Morita[1], Hiroharu Yui[2]*, Shu-hei Urashima[2], Morihiko Onose[1], Shintaro Komatani[1], Izumi Nakai[3], Yoshinari Abe[4], Yasuko Terada[5], Hisashi Homma[6], Kazuko Motomura[7], Kiyohiro Ichida[1], Tetsuya Yokoyama[8], Kazuhide Nagashima[9], Jérôme Aléon[10], Conel M. O'D. Alexander[11], Sachiko Amari[12,13], Yuri Amelin[14], Ken-ichi Bajo[15], Martin Bizzarro[16], Audrey Bouvier[17], Richard W. Carlson[11], Marc Chaussidon[18], Byeon-Gak Choi[19], Nicolas Dauphas[20], Andrew M. Davis[20], Wataru Fujiya[21], Ryota Fukai[22], Ikshu Gautam[8], Makiko K. Haba[8], Yuki Hibiya[23], Hiroshi Hidaka[24], Peter Hoppe[25], Gary R. Huss[9], Tsuyoshi Iizuka[26], Trevor R. Ireland[27], Akira Ishikawa[8], Shoichi Itoh[28], Noriyuki Kawasaki[15], Noriko T. Kita[29], Kouki Kitajima[29], Thorsten Kleine[30], Sasha Krot[9], Ming-Chang Liu[31], Yuki Masuda[8], Frédéric Moynier[18], Ann Nguyen[32], Larry Nittler[11], Andreas Pack[33], Changkun Park[34], Laurette Piani[35], Liping Qin[36], Tommaso Di Rocco[33], Sara S. Russell[37], Naoya Sakamoto[38], Maria Schönbächler[39], Lauren Tafla[31], Haolan Tang[31], Kentaro Terada[40], Tomohiro Usui[22], Sohei Wada[15], Meenakshi Wadhwa[41], Richard J. Walker[42], Katsuyuki Yamashita[43], Qing-Zhu Yin[44], Shigekazu Yoneda[45], Edward D. Young[31], Ai-Cheng Zhang[46], Tomoki Nakamura[47], Hiroshi Naraoka[48], Takaaki Noguchi[49], Ryuji Okazaki[48], Kanako Sakamoto[22], Hikaru Yabuta[50], Masanao Abe[22], Akiko Miyazaki[22], Aiko Nakato[22], Masahiro Nishimura[22], Tatsuaki Okada[22], Toru Yada[22], Kasumi Yogata[22], Satoru Nakazawa[22], Takanao Saiki[22], Satoshi Tanaka[22], Fuyuto Terui[51], Yuichi Tsuda[22], Sei-ichiro Watanabe[24], Makoto Yoshikawa[22], Shogo Tachibana[52], Hisayoshi Yurimoto[15]

*** Corresponding author. E-mail address: yui@rs.tus.ac.jp (H. Yui).**

Affiliations:
[1] Analytical Technology Division, Horiba Techno Service Co., Ltd., Kyoto 601-8125, Japan.
[2] Department of Chemistry, Tokyo University of Science, Tokyo 162-8601, Japan.
[3] Department of Applied Chemistry, Tokyo University of Science, Tokyo 162-8601, Japan.
[4] Graduate School of Engineering Materials Science and Engineering, Tokyo Denki University, Tokyo 120-8551, Japan.
[5] Spectroscopy and Imaging, Japan Synchrotron Radiation Research Institute, Hyogo 679-5198 Japan.
[6] Osaka Application Laboratory, Rigaku Corporation, Osaka 569-1146, Japan.
[7] Thermal Analysis Division, Rigaku Corporation, Tokyo 196-8666, Japan.
[8] Department of Earth and Planetary Sciences, Tokyo Institute of Technology, Tokyo 152-8551, Japan.
[9] Hawaiʻi Institute of Geophysics and Planetology, University of Hawaiʻi at Mānoa, Honolulu, HI 96822, USA.
[10] Institut de Minéralogie, de Physique des Matériaux et de Cosmochimie, Sorbonne Université, Museum National d'Histoire Naturelle, Centre National de la Recherche Scientifique Unité Mixte de Recherche 7590, Institut de recherche pour le développement, Paris 75005, France.
[11] Earth and Planets Laboratory, Carnegie Institution for Science, Washington, DC, 20015, USA.
[12] McDonnell Center for the Space Sciences and Physics Department, Washington University, St. Louis, MO 63130, USA.
[13] Geochemical Research Center, The University of Tokyo, Tokyo, 113-0033, Japan
[14] Guangzhou Institute of Geochemistry, Chinese Academy of Sciences, Guangzhou, GD 510640, China.
[15] Department of Natural History Sciences, Hokkaido University, Sapporo 001-0021, Japan.
[16] Centre for Star and Planet Formation, Globe Institute, University of Copenhagen, Copenhagen K 1350, Denmark.
[17] Bayerisches Geoinstitut, Universität Bayreuth, Bayreuth 95447, Germany.
[18] Université Paris Cité, Institut de physique du globe de Paris, Centre National de la Recherche Scientifique, Paris 75005, France.
[19] Department of Earth Science Education, Seoul National University, Seoul 08826, Republic of Korea.
[20] Department of the Geophysical Sciences and Enrico Fermi Institute, University of Chicago, Chicago, IL 60637, USA.
[21] Faculty of Science, Ibaraki University, Mito 310-8512, Japan.
[22] Institute of Space and Astronautical Science (ISAS), Japan Aerospace Exploration Agency (JAXA), Sagamihara 252-5210, Japan.
[23] Department of General Systems Studies, University of Tokyo, Tokyo 153-0041, Japan.

[24] Department of Earth and Planetary Sciences, Nagoya University, Nagoya 464-8601, Japan.
[25] Max Planck Institute for Chemistry, Mainz 55128, Germany.
[26] Department of Earth and Planetary Science, University of Tokyo, Tokyo 113-0033, Japan.
[27] School of Earth and Environmental Sciences, University of Queensland, St Lucia QLD 4072, Australia.
[28] Division of Earth and Planetary Sciences, Kyoto University, Kyoto 606-8502, Japan.
[29] Department of Geoscience, University of Wisconsin- Madison, Madison, WI 53706, USA.
[30] Max Planck Institute for Solar System Research, Göttingen 37077, Germany.
[31] Department of Earth, Planetary, and Space Sciences, University of California, Los Angeles, CA 90095, USA.
[32] Astromaterials Research and Exploration Science Division, National Aeronautics and Space Administration Johnson Space Center, Johnson Space Center, Houston, TX 77058, USA.
[33] Faculty of Geosciences and Geography, University of Göttingen, Göttingen D-37077, Germany
[34] Division of Earth-System Sciences, Korea Polar Research Institute, Incheon 21990, Korea.
[35] Centre de Recherches Pétrographiques et Géochimiques, Centre National de la Recherche Scientifique-Université de Lorraine, Nancy 54500, France.
[36] School of Science and Space Sciences, University of Science and Technology of China, Anhui 230026, China.
[37] Department of Earth Sciences, Natural History Museum, London, SW7 5BD, UK.
[38] Isotope Imaging Laboratory, Hokkaido University, Sapporo 001-0021, Japan.
[39] Institute for Geochemistry and Petrology, Department of Earth Sciences, ETH Zurich, Zurich, Switzerland.
[40] Department of Earth and Space Science, Osaka University, Osaka 560-0043, Japan.
[41] School of Earth and Space Exploration, Arizona State University, Tempe, AZ 85281, USA.
[42] Department of Geology, University of Maryland, College Park, MD 20742, USA.
[43] Graduate School of Natural Science and Technology, Okayama University, Okayama 700-8530, Japan.
[44] Department of Earth and Planetary Sciences, University of California, Davis, CA 95616, USA.
[45] Department of Science and Engineering, National Museum of Nature and Science, Tsukuba 305-0005, Japan.
[46] School of Earth Sciences and Engineering, Nanjing University, Nanjing 210023, China.
[47] Department of Earth Science, Tohoku University, Sendai, 980-8578, Japan.
[48] Department of Earth and Planetary Sciences, Kyushu University, Fukuoka 819-0395, Japan.
[49] Division of Earth and Planetary Sciences, Kyoto University, Kyoto 606-8502, Japan.

[50] Earth and Planetary Systems Science Program, Hiroshima University, Higashi-Hiroshima 739-8526, Japan.

[51] Graduate School of Engineering, Kanagawa Institute of Technology, Atsugi 243-0292, Japan.

[52] UTokyo Organization for Planetary and Space Science (UTOPS), University of Tokyo, Tokyo 113-0033, Japan.

Experiments

Inductively Coupled Plasma Atomic Emission Spectroscopy for Terrestrial Carbonates

The particle of Dolomite (MV) was dissolved in 10 mL of royal water and filled to 100 mL. The solution was analyzed to quantify Mg, Ca, Mn and Fe after further dilution. The ICP-AES was performed with OPTIMA 8300 manufactured by PerkinElmer, Inc. The calibration curves were created with JCSS reference materials (available from Kanto Chemical Co., Inc. Soka factory). The cation composition was given in wt.% by the mean values of three repeated measurements.

Cation Composition by Micro-XRF

16 points for the particle were studied by micro-XRF. The measurement conditions of the X-ray tube voltage, the X-ray tube current, X-ray working distance were 30 kV, 500 μA, 1.0 mm, respectively. The measurement time for each point was 100 s. The mean value of the 16 datapoints for cation contents based on the standard FP method were used to revise the Eqs. (2,3).

Derivation of Eq. (3) in the main text

According to our previous work (ref. 13 in the main text), Ca content (C_{Ca}) is linearly related with the T and L mode wavenumbers ($\widetilde{\nu_{\mathrm{T}}}$ and $\widetilde{\nu_{\mathrm{L}}}$). Note that $\widetilde{\nu_{\mathrm{T}}}$ and $\widetilde{\nu_{\mathrm{L}}}$ are determined in cm^{-1} unit here. Suppose we have 3 standard carbonates, whose C_{Ca}, $\widetilde{\nu_{\mathrm{T}}}$, and $\widetilde{\nu_{\mathrm{L}}}$ are known as $(C_{\mathrm{Ca},1}, \widetilde{\nu_{\mathrm{T},1}}, \widetilde{\nu_{\mathrm{L},1}})$, $(C_{\mathrm{Ca},2}, \widetilde{\nu_{\mathrm{T},2}}, \widetilde{\nu_{\mathrm{L},2}})$, and $(C_{\mathrm{Ca},3}, \widetilde{\nu_{\mathrm{T},3}}, \widetilde{\nu_{\mathrm{L},3}})$ for the

standards 1, 2, and 3, respectively. Because C_{Ca} and the wavenumbers $\widetilde{\nu_{\mathrm{T}}}$ and $\widetilde{\nu_{\mathrm{L}}}$ are linearly related, the following equations

$$\widetilde{\nu_{\mathrm{T},1}}x + \widetilde{\nu_{\mathrm{L},1}}y + z = C_{\mathrm{Ca},1}$$

$$\widetilde{\nu_{\mathrm{T},2}}x + \widetilde{\nu_{\mathrm{L},2}}y + z = C_{\mathrm{Ca},2}$$

$$\widetilde{\nu_{\mathrm{T},3}}x + \widetilde{\nu_{\mathrm{L},3}}y + z = C_{\mathrm{Ca},3}$$

are satisfied. The unknown coefficients x, y, and z are thus

$$\begin{pmatrix} x \\ y \\ x \end{pmatrix} = \begin{pmatrix} \widetilde{\nu_{\mathrm{T},1}} & \widetilde{\nu_{\mathrm{L},1}} & 1 \\ \widetilde{\nu_{\mathrm{T},2}} & \widetilde{\nu_{\mathrm{L},2}} & 1 \\ \widetilde{\nu_{\mathrm{T},3}} & \widetilde{\nu_{\mathrm{L},3}} & 1 \end{pmatrix}^{-1} \begin{pmatrix} C_{\mathrm{Ca},1} \\ C_{\mathrm{Ca},2} \\ C_{\mathrm{Ca},3} \end{pmatrix} \quad \text{(S1)}$$

For the standards 2 and 3, kutnohorite (SH) and kutnohorite (W) were used both in the previous and present study. For the standard 1, a geostandard sample named JDo-1 was used in the previous study. JDo-1 is a dolostone mined at Kuzuu, Tochigi, Japan, and it is determined as a geostandard by The National Institute of Advanced Industrial Science and Technology (AIST), Japan. We used JDo-1 because its composition is accurately determined by AIST. However, we later noticed that some portion of carbonates in JDo-1 is calcite ($CaCO_3$), not dolomite ($CaMg(CO_3)_2$). In fact, Raman band assignable to calcite were sometimes obtained from JDo-1 powder as shown in Figure S4. Therefore, while C_{Ca} of JDo-1 is determined as 0.567, some of them originate in calcite so that the cation content of 0.567 cannot be related to $\widetilde{\nu_{\mathrm{T}}}$ and $\widetilde{\nu_{\mathrm{L}}}$ of dolomite in JDo-1. Therefore, in the present study,

we used dolomite (MV) as the standard 1. Dolomite (MV) was chosen because it was relatively homogeneous as shown in Figure 3 in the main text. By substituting the parameters in Eq (S1) by experimentally determined values, we obtained

$$\begin{pmatrix} x \\ y \\ x \end{pmatrix} = \begin{pmatrix} 174.103 & 296.731 & 1 \\ 162.444 & 286.094 & 1 \\ 179.662 & 286.386 & 1 \end{pmatrix}^{-1} \begin{pmatrix} 0.494 \\ 0.661 \\ 0.126 \end{pmatrix}$$

$$= \begin{pmatrix} -0.00162371 & -0.0575557 & 0.0591794 \\ 0.0957912 & -0.0309258 & -0.0648654 \\ -27.1415 & 19.1973 & 8.94425 \end{pmatrix} \begin{pmatrix} 0.494 \\ 0.661 \\ 0.126 \end{pmatrix}$$

$$= \begin{pmatrix} -0.03139 \\ 0.01867 \\ 0.419 \end{pmatrix}$$

Namely,

$$C_{\mathrm{Ca}} = -0.03139\widetilde{\nu_{\mathrm{T}}} + 0.01867\widetilde{\nu_{\mathrm{L}}} + 0.419 \qquad (3)$$

Supplementary Information Table

Table S1. Cation compositions of terrestrial carbonates for reference. The values for kutnohorite (W) and (SH) are taken from ref.14 and the one for ferroan dolomite (EM) are from ref.13 in the main text. The values in parentheses are calculated from the quantitative results by ICP-AES.

	mined location	cation composition			
		Ca	Mg	Fe	Mn
kutnohorite (W)[14]	Wissels mine, Hotazel, Kalahari manganese field, Northern Cape, South Africa	0.126 ± 0.007	0.038 ± 0.007	0.004 ± 0.001	0.832 ± 0.010
kutnohorite (SH)[14]	Sterling Hill, Ogdensburg, Franklin Mining District, Sussex County, New Jersey, USA	0.661 ± 0.024	0.032 ± 0.023	0.004 ± 0.000	0.303 ± 0.011
dolomite (MV)	Morro Velho Mine, Nova Lima, Minas Gerais, Brazil	0.399 ± 0.002 (0.398)	0.494 ± 0.003 (0.496)	0.102 ± 0.003 (0.101)	0.005 ± 0.000 (0.006)
ferroan dolomite (EM)[13]	Eagle Mine, Colorado, USA	0.593	0.273	0.018	0.133

Supplementary Information Figures

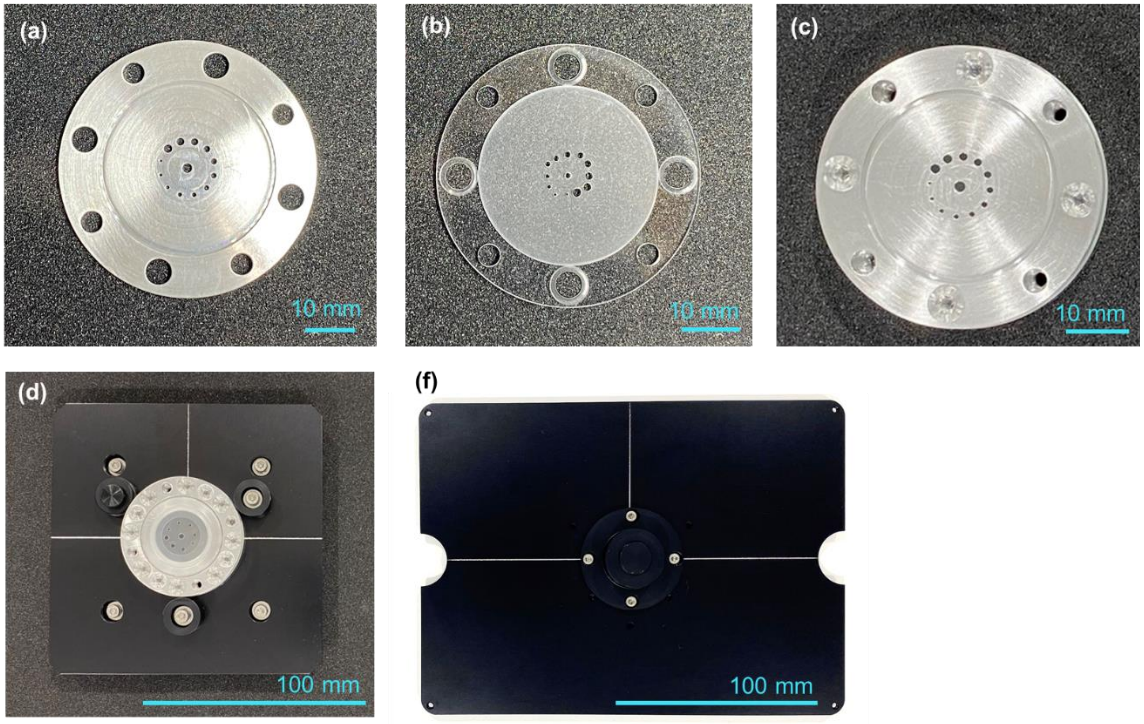

Figure S1. Optical images of Arrayed Multi-Chamber Sample Cell (AMCC). (a) Aluminum sample cell frame. (b) acrylic sample cell frame. (c) AMCC, aluminum sample cell frame built with aluminum cell frame base. (d) AMCC attached to AMCC jig for micro-XRF. (e) AMCC jig for Raman microscope.

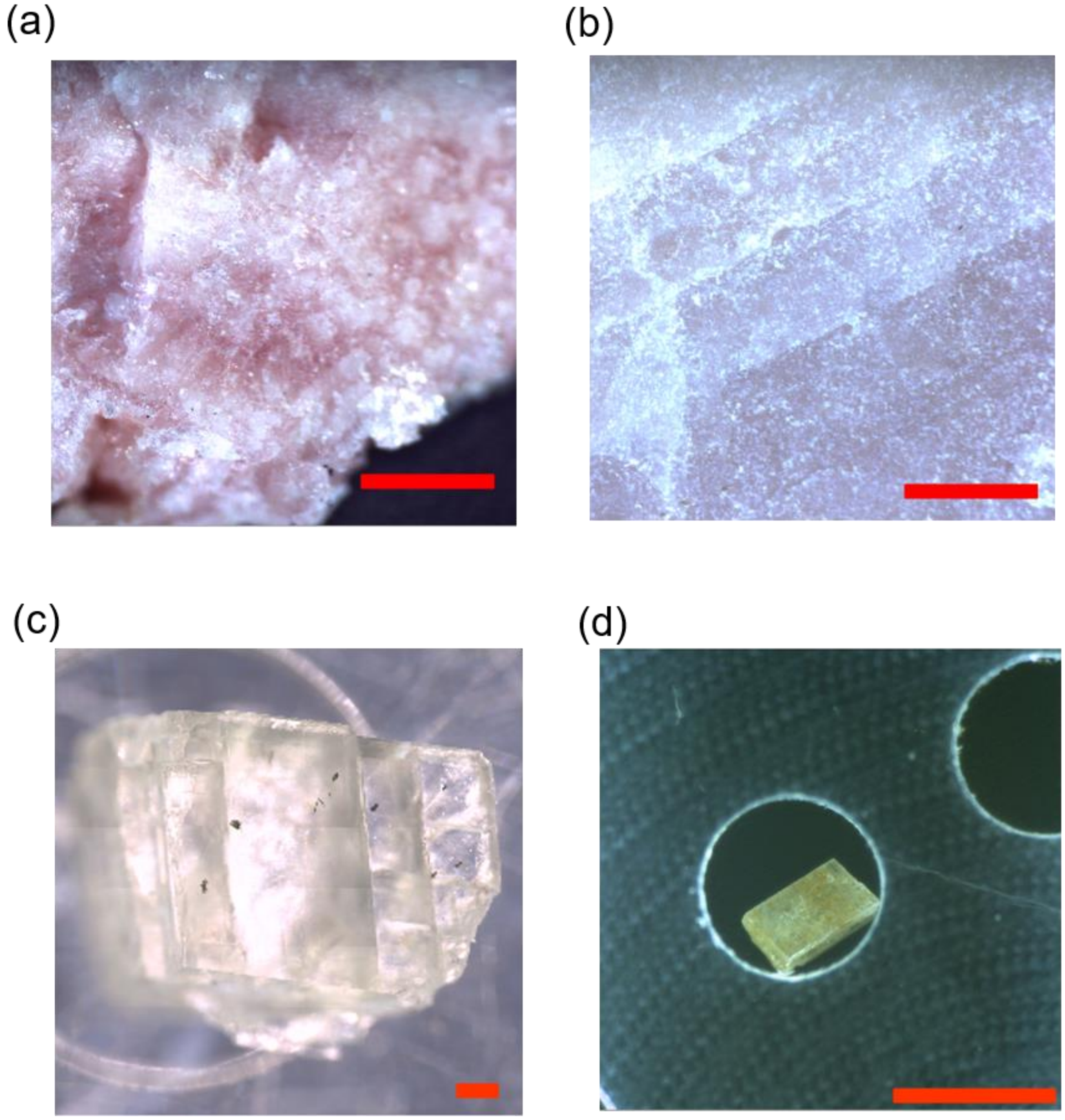

Figure S2. Optical images for terrestrial carbonates (scale bar: 1 mm). (a) Kutnohorite (W), (b) Kutnohorite (SH), (c) Dolomite (MV) and (d) Ferroan Dolomite (EM).

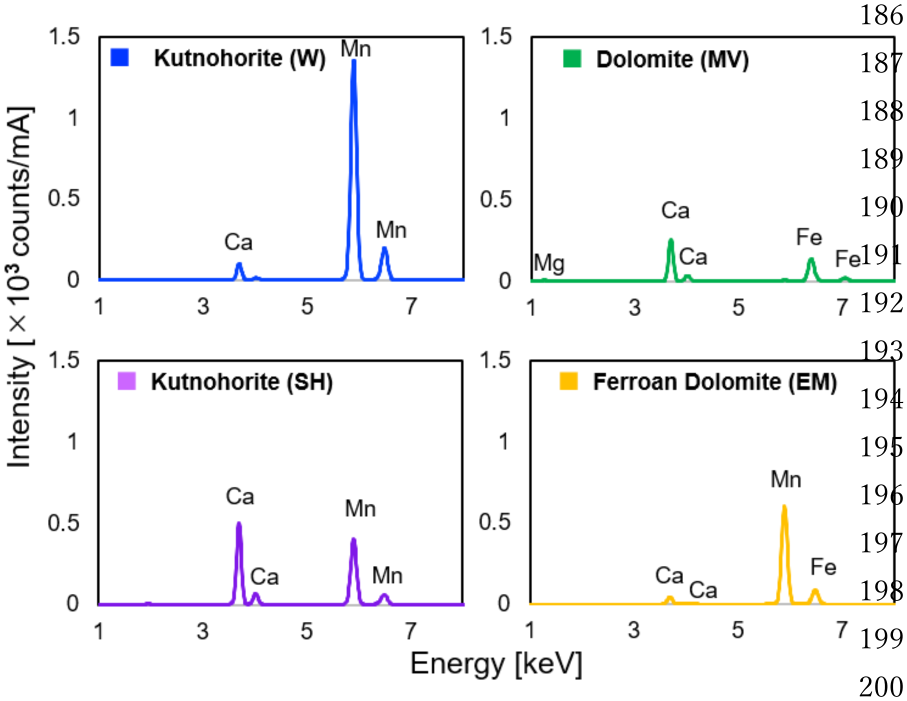

Figure S3. X-ray spectra for terrestrial carbonates.

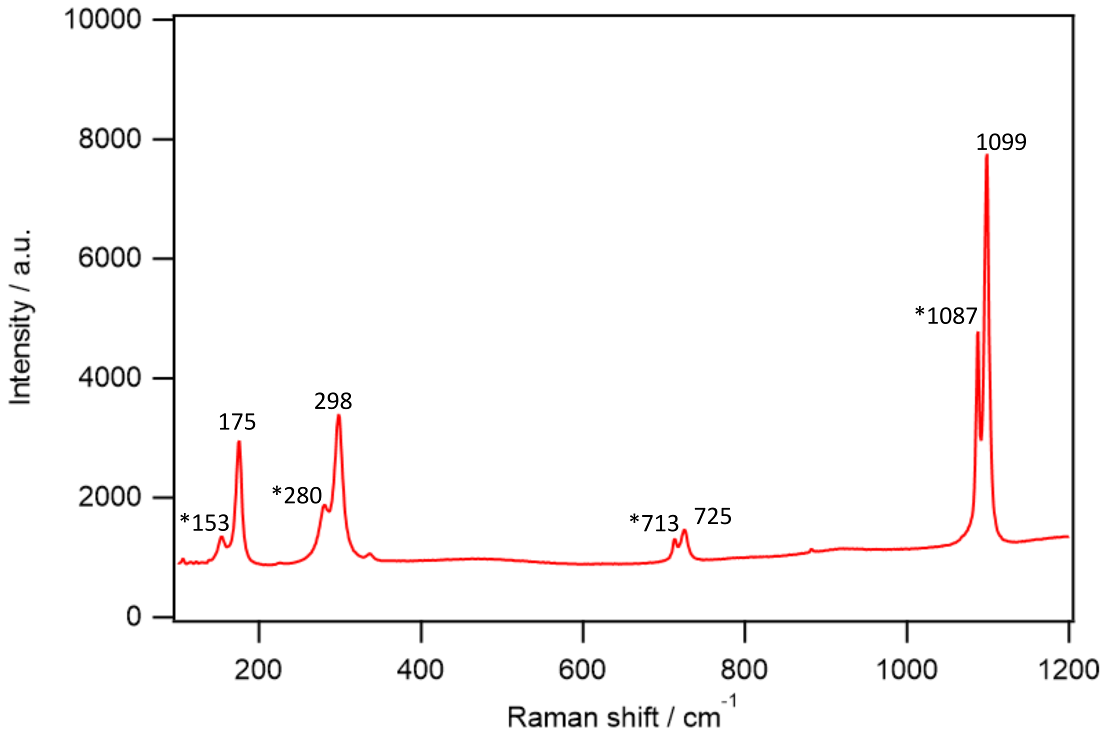

Figure S4. A Raman spectrum of JDo-1. The peaks with asterisks(*) are assignable to calcite.

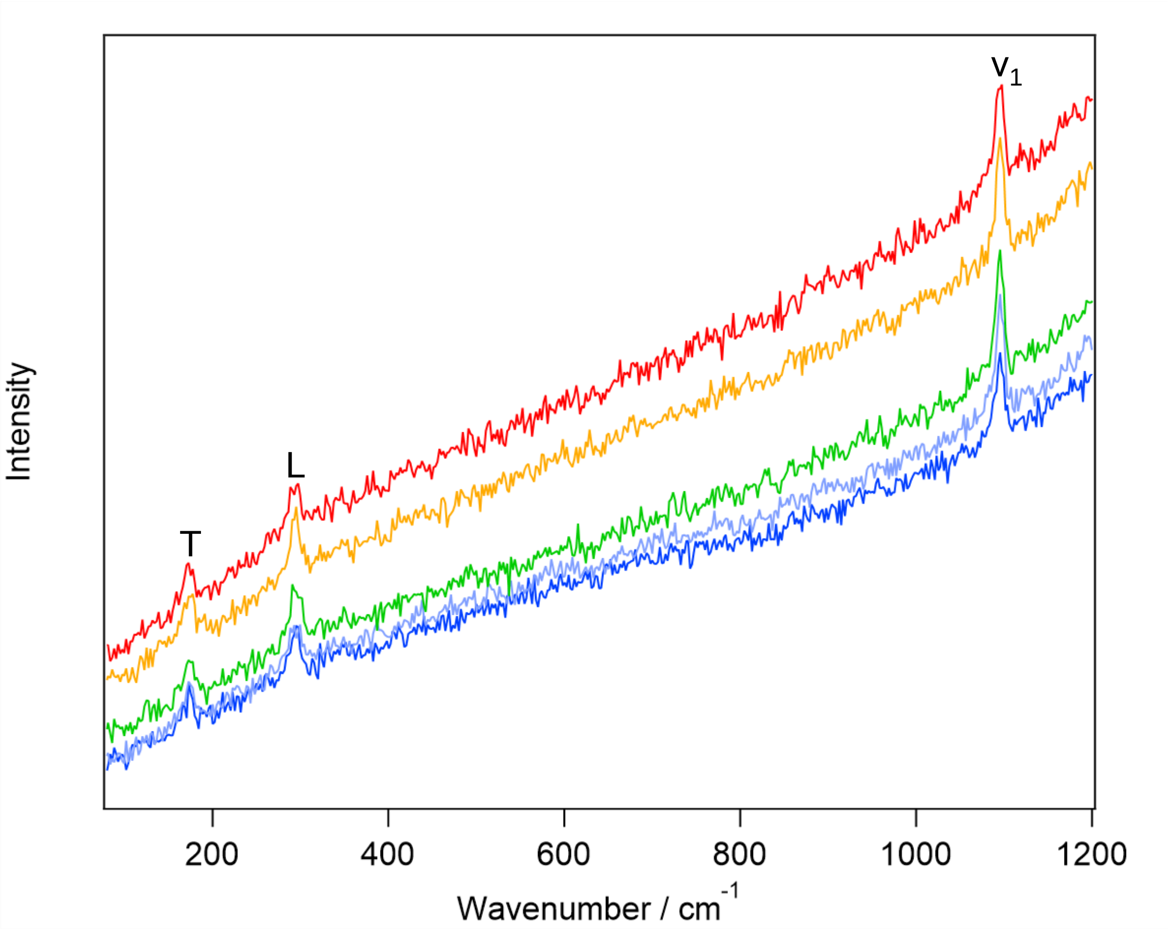

Figure S5. Raman spectra obtained at several spots in grain B.
